# Does language make people more prosocial? The influence of Mandarin proficiency on donation behavior

**DOI:** 10.3389/fpsyg.2022.1109774

**Published:** 2023-01-12

**Authors:** Qiang Wang, Laihui Yu, Ruilin Zhang

**Affiliations:** ^1^School of Liberal Arts, Jiangxi Science and Technology Normal University, Nanchang, China; ^2^School of Humanities and Communication, Guangdong University of Finance and Economics, Guangzhou, China; ^3^School of Foreign Languages, Guangzhou Huashang College, Guangzhou, China

**Keywords:** language, prosocial, Mandarin proficiency, donation behavior, perceived social responsibility, subjective social status

## Abstract

Both the effects of language proficiency on individual outcomes, and the influencing factors of individual donation arouse wide concern, but researchers have hardly probed into the relationship between language proficiency and donation behavior. Using the data from the 2012 China General Social Survey (CGSS) and the binary logistic regression model as the benchmark model, this study empirically examines the influence and mechanism of language proficiency on donation behavior. It is revealed that Mandarin proficiency has significant positive influence on individual donation behavior. According to the results of the variable substitution practice and the instrumental variable regression based on the two-stage least square model, the above conclusion remains robust. The heterogeneity test shows that Mandarin proficiency of male, southern and rural residents has more obvious impact on donation behavior. The multiple intermediary effect test indicates that perceived social responsibility and subjective social status partially mediate the relationship between Mandarin proficiency and donation behavior. Therefore, it indicates that language proficiency has a prosocial effect, which makes people more prosocial. This study contributes to the literature on donation behavior by examining the influencing mechanism of Mandarin proficiency on individual donation, and further the effects of language proficiency on individual outcomes, thereby providing theoretical and empirical support for the formulation of policies for the promotion of Mandarin and social donation in China.

## 1. Introduction

Donation is a kind of prosocial behavior that refers to helping others without a defined goal and offering tangible or intangible property to specific organizations or individuals without asking for any return (Taute and McQuitty, 2015). Individual donation has always been an important factor in promoting social fairness and justice, maintaining social harmony and stability. The level of resident giving behavior is the cornerstone of charities and one of the key reflections of the harmonious evolution of society. China has been known as a “country of etiquette” with a fine tradition of showing benevolence and taking pleasure in helping others since ancient times. As the per capita income of Chinese people keeps growing and the national legal protection for individual donations is strengthened, the Chinese public has been involved in charities with increasing enthusiasm. According to the *2019 Charitable Donation Report of China*, China received 170.144 billion RMB in domestic and international donations in 2018, with individual donations accounting for 23.4% of the total donations and representing an increase of 10.54%. Even so, there is still a significant disparity between the degree of individual donation in China and those in Western nations. Therefore, it has become crucial to investigate the variables that may influence an individual's contribution behavior and then implement necessary changes to raise the donation rate of Chinese individuals' contributions.

From a social perspective, language plays a crucial role in the development and operation of human society. It has numerous advantageous characteristics and incorporates a wide variety of functions, such as communication, culture, and society. From an individual perspective, as a comprehensive manifestation of basic human abilities and qualities, language proficiency has a substantial impact on one's human capital, social capital, and cultural capital. There are consequently consequences on aspects such as the income spillover effect (Chiswick and Miller, 1995), health effect (Schachter et al., 2012), and happiness effect (Angelini et al., 2015). The majority of study to date has focused on how linguistic competence influences individuals' capacity for economic and social advancement. Researchers have hardly looked at how linguistic ability affects people's prosocial behavior, just like whether it improves the frequency of charitable giving. Furthermore, while there are lots of linguistics-based researches on the influencing factors of individual donations, researchers have rarely probed into the relationship between language proficiency and donation behavior. Given all this, using data from the 2012 China General Social Situation Survey, this study focuses on the influence and mechanism of language proficiency on individual donation behavior.

## 2. Literature review

### 2.1. Donation behavior

The fundamental characteristic of prosocial behavior is that the provider demonstrates prosocial qualities by putting others' needs ahead of his own interests. The development of society and the economy, as well as the growing awareness of social philanthropy, are all contributing to a steady increase in public donations. The government and all levels of society have taken notice of this (Halfpenny and Lowe, 1994). The factors that affect individual donation behavior have attracted the attention of many researchers on a global and local scale. Based on reviewing more than 500 related works, Bekkers and Wiepking (2010) has classified the factors and mechanisms influencing individual donation behavior into eight categories: awareness, donation information, costs and benefits, altruism, reputation, psychological consequences, values, and efficacy. In general, academic circles look at charitable giving from the perspective of individualism and structuralism. The structuralism viewpoint stresses how structural pressures in an individual's external environment, such as their family environment, organizational environment, social environment, and institutional environment, shape their donating behavior (Yen, 2002; Wiepking and Maas, 2009; Meer, 2011; Brown and Ferris, 2016). While individualism focuses on the intrinsic factors influencing donation behavior, In addition to demographic characteristics such as gender, education, and social and economic status (Andreoni and Vesterlund, 2001; Bénabou and Tirole, 2006), psychological factors such as selfishness and altruism (Glazer and Konrad, 1996; Vesterlund, 2006), sympathy (Clary and Snyder, 1991), regrets and guilt (Dawson, 1988) are all taken into consideration from an individualistic perspective, which emphasizes the inner role of donors. According to other academics, a person's donation does not entirely depend on good intentions or altruism, but also on resources such as material and time (Penner et al., 2005) as well as their ability and willingness to make donations (Korndorfer et al., 2015).

### 2.2. Language proficiency and prosocial behavior

As an integral part of human capital, language skills have a profound impact on individuals' economic and social lives. According to linguistic economics, languages proficiency is a vital part of human capital in the context of the economy (Chiswick and Miller, 2003). People with stronger language skills have more communication alternatives, are better able to make more money or have a higher quality of life (Gao and Smyth, 2011), and receive higher income or get better jobs (Chiswick and Miller, 1995; Lazear, 1999; Lawson and Sachdev, 2016). Language proficiency is integrally tied to a person's success in the labor market, including income levels, employment opportunities, and particular jobs (Dustmann, 1994; Chiswick and Miller, 1995). Language carries the dissemination of knowledge and the transfer of culture (Bourdieu and Passeron, 1990). Language as a social bond that brings different individuals together into a community and weave a social network (Zhao, 2013). People with high language proficiency are more concerned about social dynamics, participate in social activities, engage in public services and expand social interactions, which leads to a closer connection with society, thus reinforce the tendency to be more prosocial, and thus more likely to engage in prosocial behavioral activities such as donations (Lu et al., 2018). Developmental psychology research has shown that language ability is one of the indicators to gauge and predict prosocial behavior, and they also show a positive relationship between children's language capability and prosocial behavior (Cassidy et al., 2003). In summary, it can be inferred that those who have the greater linguistic ability are more likely to acquire greater survival or social skills, engage in more social interactions, have a stronger sense of social identity, and thus are more willing to make donations. As a result, the following research supposition is made in this study.

*H1: Individual donation behavior is significantly increased by language proficiency*.

### 2.3. Mechanisms: Altruism and reciprocity

The issue of why individuals choose to make donations is a topic that has long been of interest (Wispé, 1978). Different scholars have provided a wide range of opinions on the motivation of donation behavior and have constructed their own theoretical foundations, and the basic view is that the act of donating is altruistic, but also self-interested (Akerlof and Kranton, 2000; DellaVigna et al., 2012). We attribute human giving behavior to a combination of two main types of motivations: one is driven by other-oriented altruism, purely to meet the needs of others (Becker, 1974; Andreoni, 1989, 1990); the other is driven by self-oriented egoism, such as to gain a high social status, a good personal reputation, or the hope of reciprocity (Sugden, 1984; Glazer and Konrad, 1996; Bénabou and Tirole, 2006). In view of this classification, some factors are expected to moderate the relationship between language proficiency and donation behavior from the mechanism of altruism and reciprocity.

Altruism refers to a psychological motivation that focuses on the interests of others without considering one's own interests; it is a level of motivation that is voluntary and explicitly committed to helping others (Schlosser and Levy, 2016). One of the main manifestations of an individual's altruistic motivation is a sense of social responsibility, which is the tendency of individuals to voluntarily work for the prosperity of society and the common good of its members with the aim of altruism (Starrett, 1996). Perceived social responsibility is more of an internalized value, which individuals believe they have a responsibility to contribute to society and make it a better place to live. Thus, responsibility is a code of conduct that individuals adhere to for themselves, and individuals are driven to engage in prosocial behavior through their own sense of social responsibility. Therefore, perceived social responsibility is a crucial factor in predicting an individual's prosocial behavior (Berkowitz and Daniels, 1963), and the level of prosocial behavior of individuals depends on the amount of social responsibility activated by situational and individual factors (Schwartz, 1977). It has been shown that people who value social responsibility make more charitable donations (Schuyt et al., 2010). On the other hand, language serves as a social link and a system or convention throughout society (Zhao, 2013). Language plays a crucial role in the development of cultural identity, individual identity, and community. It also plays a crucial role in the web of meaning that people weave in their lives. People who share the same language come together to form an indivisible whole because of the social aspect of language, which states that “those who speak the same language have an intangible and strong attraction” (Fichte, 2008). Therefore, raising people's language proficiency can increase their desires to engage in society, foster interpersonal contacts, and strengthen perceived social responsibility, which is shown in their participation in charitable activities and public services (Lu et al., 2018). This suggests that having a strong command of a second language helps one build a larger social network, develop a stronger sense of civic duty, and become more willing to engage in and put into practice charitable activities. In light of this, this study suggests the following research hypothesis.

*H2: Language proficiency has an impact on promoting one's donations through heightening their perceived social responsibility*.

Social exchange theory and the reciprocity perspective suggest that people are more likely to engage in prosocial behaviors when they are rewarded or reciprocated by the recipient (Leimgruber, 2018). In other words, people are more likely to help others if the (potential) rewards of the relationship outweigh the costs/investment. In this context, helping others is often motivated by the expectation of establishing and maintaining a positive relationship with the target, and individuals tend to build/maintain relationships and promote interpersonal harmony. Thus, the individual tends to engage in prosocial behavior in order to get what he needs from others as well. For example, to following social norms, building a good self-image and win a high reputation are the motives that induce individual donation behavior (Sugden, 1984; Whillans and Dunn, 2018). Compared to lower class individuals, higher class individuals are more concerned about their reputation and group identity and have a greater desire for class (Belmi et al., 2020), so they are more likely to follow prosocial norms and produce more prosocial behavior. It can be inferred from this that in order to maintain their good image and enhance their reputation, individuals with higher subjective class identity are more likely to perform donation behaviors. On the other hand, sociolinguistics contends that language can reflect the social class of the speaker, that the speaker's response to particular linguistic variables is compatible with his socioeconomic class, and that there are glaring variations in language use across social classes. The language skills of children from wealthy families and those from lower-class families differ significantly (Bernstein and Henderson, 1969). Language changes correspond to changes in one's identity, and identity changes have a significant impact on one's attitudes toward languages and language use (Lawson and Sachdev, 2016). An individual's sense of class identification is strengthened by language proficiency, an essential component of cultural capital. It can be concluded that those who are more linguistically skilled are more likely to feel more strongly about their class, which makes them more likely to give to charity. Therefore, this study suggests the following research hypothesis.

*H3: Language proficiency has an impact on promoting one's donations through enhancing their subjective social status*.

### 2.4. Theoretical framework

**Figure 1:**
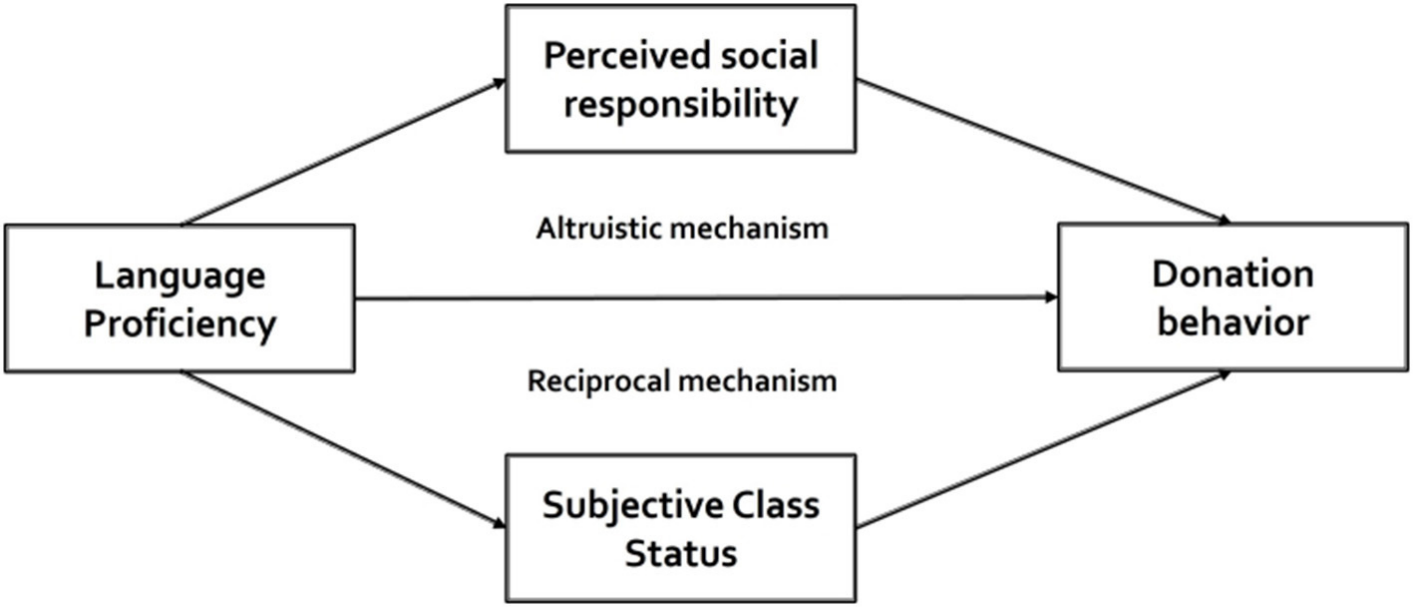
The theoretical framework.

Based on the above analysis, combining social identity theory, social norm theory, social exchange theory, the theoretical framework of this study is constructed (Figure 1). The framework explains how individuals' language proficiency affects their donation behavior. Specifically, language proficiency promotes ones' donation behavior, mainly through subjective social status and perceived social responsibility, which respectively stand for altruistic and reciprocal mechanisms.

## 3. Research method

### 3.1. Data sources

The 2012 China Comprehensive Social Status Survey (CGSS), which was carried out by the Renmin University of China in collaboration with academic institutions nationwide, provided the data for this study. The research is the country's first comprehensive, ongoing, and national academic survey project. It employs a multi-stage stratified random sampling design and gets data from 29 provinces, autonomous areas, and municipalities nationwide, which completely and methodically gathers data on social life, including politics, economics, and culture in China, and has a high validity, and whose data has been used in a great number of empirical research. It originates at the social, family, and individual levels. There are two volumes (Volume A and Volume B) to the CGSS2012 questionnaire. The variables needed for this study are included in Volume A's data, which also includes a total of 5,819 samples. 5,171 valid data were finally collected after the data had been cleaned up, and invalid and outlier data were deleted.

### 3.2. Description of variables

#### 3.2.1. Dependent variable: Donation behavior

The dependent variable of this study is donation behavior. The variable is measured by asking the question, “In 2011, have you personally made a donation to society in the form of money, in kind, or ownership? In this context, we refer to donations that you have made to individuals or organizations in the community voluntarily and without the intention of receiving a donation back.” We set the value of this variable to 1 for participating donations and to 0 for non-participating donations.

#### 3.2.2. Key independent variable: Mandarin proficiency

The independent variable of this study is Mandarin proficiency. According to Zhang and Lien (2020), speaking is the key aspect of Mandarin competence. The variable is measured by asking the question, “What do you think of your ability to speak Mandarin?” measured on a 5-point Likert scale ranging from 1 (none) to 5 (Very well).

#### 3.2.3. Mediating variables: Perceived social responsibility and subjective social status

The mediating variables include perceived social responsibility and subjective social status. The variable of perceived social responsibility is measured by asking the question, “To what extent do you agree with the statement that I want to make a contribution to society?” measured on a 7-point Likert scale ranging from 1 (strongly disagree) to 7 (strongly agree). According to Chen and Williams (2018), the variable of subjective social status is measured by asking the question, “In our society, some individuals belong to the upper class of society and some people belong to the lower class of society, which class do you think you are currently in?” We set the value of this variable to 1 for the lowest class and to 10 for the highest class.

#### 3.2.4. Control variables

In terms of control variables, previous studies show that social capital has a significant effect on individuals' donations (Schervish et al., 1997; Mesch et al., 2006; Wang and Graddy, 2008; Brown and Ferris, 2016). However, those scholars' findings were inconsistent probably because of differences between Chinese and Western cultures or differences in the measurement of social capital. Therefore, we used social capital as a control variable and divided it into two dimensions, namely structural social capital and cognitive social capital (Uphoff, 2000). The variable of cognitive social structure is measured by asking the question, “How often do you engage in social and recreational activities with your friends (for example, visiting each other, watching TV, having a meal, playing cards, etc.)” We set the value of this variable ranging from 1 (never) to 7 (almost every day). The variable of cognitive social structure is measured by asking the question, “In general, to what extent do you think most people can be trusted?” We set the value of this variable ranging from 1 (Dealing with most people almost always requires great care) to 4(Most people can almost always be trusted). According to the existing literature (Andreoni and Vesterlund, 2001; Bénabou and Tirole, 2006), we also considered demographic, economic, and social factors, and included control variables such as gender, age, marriage, ethnicity, religion, party membership, household registration, residence, years of education, and income levels, which are shown in the descriptive statistics in Table 1.

**Table 1:** The method of variable measurement and the result of descriptive statistics (*N* = 5,171).

**Variable**	**Definition**	**Mean**	**SD**	**Min**	**Max**
Donation behavior	Donation = 1, non-donation = 0	0.33	0.470	0	1
Mandarin speaking	None = 1, bad = 2, moderate = 3, well = 4, very well = 5	3.09	1.208	1	5
Mandarin speaking	Great = 1, poor = 0	0.35	0.477	0	1
Gender	Male = 1, female = 0	0.51	0.500	0	1
Age	Age/100	48.27	16.082	16	93
Marriage	Married = 1, others = 0	0.81	0.395	0	1
Ethnicity	Han nationality = 1, others = 0	0.91	0.285	0	1
Religion	Have a religious belief = 1, have no religious belief = 0	0.86	0.343	0	1
Party membership	Member of the Chinese Communist Party = 1, non-party members = 0	0.12	0.331	0	1
Household registration	Resident committee = 1, village committee = 0	0.47	0.499	0	1
Residence	Urban = 1, rural = 0	0.55	0.497	0	1
Education	Never receive any education = 0, private or elementary school = 6, junior high school = 9, high school = 12, junior college = 15, bachelor = 16, graduate and above = 18	8.79	4.588	0	18
Ln (income)	Natural logarithm of personal income in 2011	3.74	1.251	0	7
Structural social capital	Frequency of social and recreational activities with friends (1–7)	4.07	1.935	1	7
Cognitive social capital	Level of trust most people (1–4)	2.97	0.614	1	4
Perceived Social Responsibility	Willingness to make a contribution to society (1–7)	5.36	1.192	1	7
Subjective social status	Self-perception of the social class (1–10)	4.17	1.688	1	10

### 3.3. Model construction

Considering that the variables employed to measure donation behavior are binary dummy variables, this research primarily uses the binary logistic model to investigate the impact of language proficiency on donation behavior. The vector of these independent variables can be written as *x'* = (*x*_1_, *x*_2_, …, *x*_*n*_), and the conditional generalization *P* (*Y* = 1 | *x*) = *p* is a donation. Then its binary Logistics regression model can be expressed as:


$$\begin{array}{l} \ln\left(x\;=\;\frac{\text{p}}{1\;-\text{p}}\right)\;=\;\beta_{0}+\beta_{1}x_{1}+\beta_{2}x_{2}+\ldots \beta_{n}x_{n} \end{array}$$

β_0_ is a constant and β_*i*_ is the coefficient of the variable of Mandarin proficiency and a series of control variables.

## 4. Empirical analyses

### 4.1. The influence of language proficiency on prosocial behaviors

#### 4.1.1. Benchmark regression

Table 2 shows the binary logistic regression model of the influence of mandarin proficiency on donation. Model (1) is a single variable regression model, and model (2) controls the control variables that affect individuals' donations. The results show that in both model (1) and model (2), the coefficient of the influence of Mandarin proficiency is positive at the significance level of 1%, indicating that Mandarin proficiency has a significant impact on prompting an individual to make donations, that is, a respondent who has greater Mandarin proficiency is more likely to donate. After Model (2) has incorporated control variables, the absolute value of the marginal effect of the influence coefficient of Mandarin proficiency decreases slightly, but it is still positive at the significance level of 1%. It suggests that Mandarin proficiency has a significant impact on individual donations behavior after the variables such as gender, age, and social capital have been controlled.

**Table 2:** Effect of Mandarin proficiency on donation behavior: Baseline regression (*N* = 5,171).

**Variables**	**1**	**2**	**3**	**4**
Mandarin speaking	0.414^***^ (0.026)	0.171^***^ (0.031)	0.256^***^ (0.070)	
Mandarin listening				0.170^***^ (0.035)
Gender		0.239^***^ (0.067)	0.242^***^ (0.067)	0.249^***^ (0.067)
Age		−0.012^***^ (0.002)	−0.013^***^ (0.002)	−0.012^***^ (0.002)
Marriage		−0.250^**^ (0.084)	−0.258^**^ (0.084)	−0.240^**^ (0.084)
Ethnicity		0.328^**^ (0.113)	0.317^**^ (0.113)	0.360^**^ (0.114)
Religion		0.478^***^ (0.093)	0.468^***^ (0.093)	0.474^***^ (0.093)
Party membership		−0.551^***^ (0.099)	−0.547^***^ (0.098)	−0.551^***^ (0.098)
Household registration		−0.202* (0.088)	−0.219* (0.088)	−0.211* (0.088)
Residence		−0.269^**^ (0.085)	−0.293^***^ (0.085)	−0.287^***^ (0.085)
Education		0.077^***^ (0.011)	0.085^***^ (0.011)	0.081^***^ (0.011)
ln (income)		0.073^**^ (0.028)	0.077^**^ (0.028)	0.076^**^ (0.028)
Structural social capital		−0.001 (0.017)	0.002 (0.017)	0.000 (0.017)
Cognitive social capital		0.149^**^ (0.052)	0.151^**^ (0.052)	0.148^**^ (0.052)
Constant	−2.028^***^ (0.091)	−1.611^***^ (0.322)	−1.194^***^ (0.310)	−1.742^***^ (0.334)
Nagelkerke R^2^	0.071	0.161	0.157	0.159
Observed values	5,171	5,171	5,171	5,171

^***^p < 0.001,

^**^p < 0.01,

^*^p < 0.05. Standard errors are in parenthesis.

In terms of the control variables, the regression coefficient of gender is significantly positive, indicating that males are more likely to donate than females. The age regression coefficient is significantly negative, indicating that an individual becomes less likely to donate as they grow older. The regression coefficient of marriage is significantly positive, indicating that married people are more likely to donate than unmarried people. People who have Han nationality are more likely to donate than ethnic minorities. Religious beliefs have a significant effect on donation, and those who hold religious beliefs are more likely to donate. Members of the Communist Party of China are more likely to donate than other groups. Residents who hold urban household registration are more likely to donate than those who hold rural household registration. The regression coefficient of education is significantly positive, indicating that those who are well-educated are more likely to donate than those who are ill-educated. In terms of the economic factors, the influencing coefficient of the income logarithm is significantly positive, indicating that those who are well-paid are more likely to donate than those who are badly paid, which confirms the hypothesis about the relationship between resources and prosocial behaviors, that is, individuals on a higher income have more resources needed for the donation and, as a result, are more likely to make donations. In terms of social factors, different types of social capital have different effects on donation. Cognitive social capital has a significant and positive influence on individuals' donation, while structural social capital does not have a significant effect, suggesting that individuals' donation depends more on their subjective cognitive social capital rather than their objective structural social capital.

#### 4.1.2. Robustness check

We used different research methods and the variable substitution practice to test the robustness of the effect that Mandarin proficiency has on donation. Firstly, Model (3) in Table 2 is the result obtained after we have set Mandarin proficiency as a dummy variable, assigned 1–3 as 0 and 4–5 as 1, and conducted the binary logistic regression analysis. The results are consistent with those of Model (2), that is, the coefficient of Mandarin proficiency is significantly positive. Therefore, we obtained the same results by using different research methods, that is, Mandarin proficiency has an impact on prompting individuals to make donations. It suggests that the effect of Mandarin speaking on individuals' donations is robust.

Secondly, as another important measure of Mandarin proficiency, listening can also represent individuals' proficiency in the language, so we replaced the independent variable Mandarin proficiency with another one, namely Mandarin listening proficiency, and conducted the binary logistic regression analysis again. The results of model (4) in Table 2 show that the coefficient of listening proficiency in Mandarin is still significantly positive and is still significant at the significance level of 1%. In other words, Mandarin listening also has a significant positive effect on individuals' donations and is also a predictor variable of individuals' donations. Significantly, the regression coefficient of Mandarin speaking is larger than that of Mandarin listening, indicating that the former has a greater impact on individuals' donations than the latter. It is because, compared to listening proficiency, Mandarin speaking proficiency plays a greater role in daily life, workplaces, and social interactions. Those who show greater Mandarin speaking proficiency are more proactive in social interactions, more likely to obtain various resources, and thus more likely to donate.

#### 4.1.3. Endogenous analysis

In the baseline regression, we, by using the logistic model, have found that there is a positive relationship between Mandarin proficiency and individuals' donation, and the finding did not change after a series of robustness checks. However, the logistic estimation was not sufficient to prove that there is a necessary causal relationship between them, so we needed to further resolve the endogenous problems caused by the omission of variables, measurement errors, and reciprocal causality. Therefore, we used instrumental variables and two-order least squares (2SLS) for further tests. Imitating the methods used by Zhang and Cheng (2022), we used the frequency of newspaper use and the average level of people's Mandarin proficiency in one city as instrumental variables. First, since only standard Mandarin is available in the newspapers, frequently reading newspapers may have a positive effect on people's Mandarin proficiency, can enlarge their vocabulary, and thus increase their speaking proficiency. Therefore, the frequency of newspaper use indicates respondents' mastery of standard Mandarin, and we used it as the first instrumental variable in this study. Second, languages vary from region to region, and the average level of respondents' Mandarin proficiency in one city can reflect their true language proficiency but is not related to their donation. Therefore, the average level of other peoples' Mandarin proficiency in the same city (excluding the respondents) is used as the second instrumental variable in our study.

The results of the data analysis (see Table 3) show that, when the variables such as other demographic variables, income levels, and social capital have been controlled, the regression coefficients of the effect of Mandarin proficiency on the frequency of newspaper use and the average level of Mandarin proficiency of other people in the same city are 0.076 and 0.699 respectively in the first-stage regression analysis, indicating that the frequency and the average level have a significant and positive effect on individuals' Mandarin proficiency, and both are significant at the 1% level. Therefore, newspaper use and Mandarin proficiency of other people in the same city are valid instrumental variables, and the latter is a more powerful instrumental variable. The results of the second-stage regression analysis show that after controlling potential endogenous problems in the model, Mandarin proficiency still has a positive effect on donation and is significant at the 1% level, indicating that Mandarin proficiency has a significant impact on promoting individuals' donation.

**Table 3:** Effect of language proficiency on donation behavior: endogenous analysis/instrumental variable method (*N* = 5,171).

**Variable name**	**Donation behavior**	
	**The first-stage regression analysis**	**The second-stage regression analysis**
Mandarin proficiency		0.033^***^(0.006)
Newspaper use	0.076^***^(0.012)	
Mandarin proficiency of other people in the same city	0.669^***^(0.017)	
Control variable	Yes	Yes
F test	334.013	51.685
Observed value	5,171	5,171

^***^p < 0.001, ^**^p < 0.01, ^*^p < 0.05. Standard errors are in parenthesis.

#### 4.1.4. Heterogeneity analysis

Given that the effect of Mandarin proficiency on donation varies from group to group, we conducted grouped logistic regression analysis of the samples from the perspectives of gender, town and country, and region. The results are shown in Table 4. Firstly, existing studies show that there are differences between men and women in terms of language learning and language proficiency (Newman et al., 2008; Coates, 2015), and the differences may lead to variations in the effects of Mandarin proficiency on donation. Therefore, we grouped the samples according to gender to examine the differences in the effect of Mandarin proficiency on male and female residents' donations. Model (5) and Model (6) are the results of the regression analysis of the effect of Mandarin proficiency on male and female residents' donation respectively. The results of data analysis show that although the significance of the regression coefficients of each equation decreases compared to the results in Table 2, they are still significant at the 5% level, indicating that Mandarin proficiency has a significant effect on both male and female residents' donations. In terms of the regression coefficients, the coefficients of males are larger than those of females, in other words, Mandarin proficiency has more obvious impact on promoting male residents' donation. It is probably because, compared with female residents, the odds are greater that Mandarin proficiency will become a barrier to male residents participating in social activities, and consequently to reduce the chance of participating donations.

**Table 4:** Effects of Mandarin proficiency on donation behavior after grouping (*N* = 5,171).

**Variable name**	**5**	**6**	**7**	**8**	**9**	**10**
	**Male**	**Female**	**Urban**	**Rural**	**Northern**	**Southern**
Mandarin proficiency	0.099^***^ (0.099)	0.068^**^ (0.009)	0.047* (0.009)	0.124^***^ (0.008)	0.046* (0.009)	0.101^***^ (0.009)
**The first-stage estimation–Mandarin speaking**						
Newspaper use	0.075^***^ (0.016)	0.092^***^ (0.018)	0.070^***^ (0.014)	0.100^***^ (0.022)	0.045* (0.018)	0.135^***^ (0.015)
Mandarin proficiency of other people in the same city	0.426^***^ (0.024)	0.428^***^ (0.025)	0.428^***^ (0.022)	0.437^***^ (0.028)	0.543^***^ (0.025)	0.215^***^ (0.028)
Control variable	yes	yes	yes	yes	yes	yes
F test	29.146	28.327	22.966	18.375	21.703	30.804
Observed value	2,653	2,518	2,864	2,307	2,366	2,805

^***^p < 0.01,

^**^p < 0.05,

^*^p < 0.10. Standard errors are in parenthesis.

Secondly, there are obvious barriers and different levels of social and economic development between urban and rural areas in China, causing different popularity of Mandarin in both areas. Therefore, differences between urban and rural residents in terms of Mandarin proficiency may lead to different impacts that Mandarin proficiency has on donation. Therefore, we grouped the samples according to urban and rural areas to examine the differences in the effects of Mandarin proficiency on urban and rural residents' donations. Models (7) and (8) show the results of regression analysis of the effect of Mandarin proficiency on urban and rural residents' donations. The results of the data analysis show that rural residents' Mandarin proficiency has a greater positive effect on their donation than urban residents. It is because rural residents' Mandarin proficiency is significantly lower than urban residents, which inhibits their productive capacity and ability to live. Therefore, increasing rural residents' Mandarin proficiency can make them more likely to donate.

Finally, China is a vast country, and there is a big difference between north China and south China in terms of language use. The differences between southern and northern residents in terms of their Mandarin proficiency may lead to differences in the effects of Mandarin proficiency on donation. Therefore, we grouped the samples according to regions, used the Qinling Mountains and Huai River as the boundary between north China and south China, and examined the effect that Mandarin proficiency has on the donation of southern and northern residents. The results of the data analysis show that the coefficient of Mandarin proficiency of southern residents is larger than that of northern residents, and has a greater positive effect on their donation. It is because there is a significant difference in language between the north and the south of China. Southern residents have difficulty in speaking Mandarin, so it will inhibit them from communicating and adapting to the changing environment and has a negative effect on their donation behavior.

### 4.2. The mechanism by which language proficiency affects prosocial behaviors

Based on the discussion in the theoretical section, we have investigated the mechanism by which reading affects prosocial behaviors. We selected perceived social responsibility and subjective social status as mediator variables and used the bias-corrected percentile Bootstrap to test the intermediary effects. In terms of testing tools and working methods, we used the SPSS macro-PROCESS plug-in. Model (4) is chosen. We, after controlling relevant variables such as demographics, economic factors, and social capital, have tested the intermediary effects of perceived social responsibility and subjective social status on language proficiency and donation, respectively.

As shown in Table 5, the results of the regression analysis show that Mandarin proficiency has a positive and significant effect on perceived social responsibility and subjective social status, and perceived social responsibility and subjective social status also have a positive and significant effect on donation statistically. The results of 5,000 Bootstrap sampling tests (95% confidence interval) show that Mandarin proficiency has a direct effect on donation (Effect = 0.160, SE = 0.032, LLCI = 0.098, ULCI = 0.222), and the confidence interval of indirect effects of perceived social responsibility (Effect = 0.008, SE = 0.003, LLCI = 0.002, ULCI = 0.015) and subjective social status (Effect = 0.004, SE = 0.002, LLCI = 0.001, ULCI = 0.009) do not contain 0. It suggests that both perceived social responsibility and subjective social status exert partial intermediary effects on Mandarin proficiency and donation (see Table 5). In other words, not only does Mandarin proficiency have a direct effect on individuals' donation behavior, but it also has an indirect effect on donation behavior through the mediation of perceived social responsibility and subjective social status.

**Table 5:** The influencing mechanism of Mandarin proficiency on donation behavior (*N* = 5,171).

**Effect**	**BC 95% CI**				
**Direct Effect**	**Estimate**	**S.E**.	***p***	**LLCI**	**ULCI**
Mandarin proficiency–donation behavior	0.160	0.032	0.000	0.098	0.222
Mandarin proficiency–perceived social responsibility	0.044	0.016	0.007	0.012	0.076
Mandarin proficiency–subjective social status	0.068	0.0230	0.003	0.023	0.113
Perceived social responsibility–donation behavior	0.182	0.028	0.000	0.126	0.237
Subjective social status–donation behavior	0.058	0.019	0.003	0.020	0.096
Total indirect effect	0.012	0.004	—	0.005	0.020
Mandarin proficiency–perceived social responsibility–donation behavior	0.008	0.003	—	0.002	0.015
Mandarin proficiency–subjective social status -donation behavior	0.004	0.002	—	0.001	0.009

All of the control variables were controlled.

In terms of direct effects, the results of data analysis show that Mandarin proficiency has a significant and positive effect on perceived social responsibility and subjective social status, that is, greater Mandarin proficiency makes people feel a stronger sense of perceived social responsibility and subjective social status. On the one hand, language, as a kind of human capital and social capital, is an important part of individuals' abilities and endowment. As the old saying goes, “those who have the greater ability should shoulder greater responsibility”. Therefore, language will heighten individuals' sense of perceived social responsibility; on the other hand, language, as a kind of cultural capital, is an important condition for individuals to be recognized by the upper class, and language proficiency helps to heighten their sense of subjective social status. In addition, both perceived social responsibility and subjective social status have a significant and positive effect on individuals' donations. Those who feel a stronger sense of perceived social responsibility are more likely to make donations. It is because perceived social responsibility, as a set of internalized values, is closely related to prosocial behaviors, and as a person believes that he has the greater responsibility to make contributions to society, they will more likely to make donations. Subjective social status has a significant and positive effect on donation. As an individual rates their social class more highly, they will more likely to make donations. It is probably because individuals who have a stronger sense of subjective social status will be more satisfied with their lives, believing in the idea of “those who get ahead in their careers should make contributions to society”, and, as a result, will be more likely to make donations.

## 5. Research discussion

### 5.1. Conclusions

Language proficiency is a fundamental skill that everyone must possess and is crucial to day-to-day living. The greater a person's language proficiency is, the simpler it may be for them to establish a larger feeling of social duty and subjective social status, which makes it simpler for them to engage in and carry out prosocial actions like giving. The relationship between language ability and charitable conduct has not yet developed a systematic and clear cognition, and it is unclear whether language can make individuals prosocial or whether language skill has a prosocial effect. Based on the 2012 CGSS data, this study thoroughly used benchmark regression (binary logistic regression), robustness test, endogeneity test, heterogeneity analysis, and mediation effect test to empirically investigate the influence and mechanism of Mandarin proficiency on donation behavior among residents in China. The following conclusions are made.

First, the benchmark regression results showed that Mandarin proficiency can significantly promote individuals' donation behavior. Even after controlling for gender, age, education level, social capital and other factors, language proficiency still has a significant impact on donation behavior. Both Mandarin speaking and listening considerably increase the level of individual donating behavior. The outcomes of the instrumental variable regression test demonstrate a significant impact of Mandarin proficiency on a person's willingness to give. This finding suggests that in addition to benefiting individuals themselves by helping to increase personal income and enhance subjective well-being, language proficiency has a significant prosocial effect by making people more prosocial and promoting individual levels of giving behavior. Mandarin is a tool for everyday communication among Chinese residents. The stronger an individual's Mandarin ability, the more likely he or she is to interact with others, and the stronger his or her ties to society, the more likely he or she is to develop prosocial tendencies and thus more likely to implement donation activities.

Second, the heterogeneity test revealed the differential effects of language ability on donation behavior across resident groups. Mandarin proficiency of male, southern and rural residents has more obvious impact on donation behavior. This is because compared with female, urban and northern residents, male, rural and southern residents have a lower level of Mandarin, and their difficulty in accurately listening and fluently speaking of Mandarin restricts their daily communication and social integration, which is not conducive to the formation of prosocial tendencies. Therefore, the language barrier has a more significant inhibitory effect on their donation behavior.

Finally, perceived social responsibility and subjective social status play a partially mediating effect between people's language proficiency and donation behavior, i.e., language proficiency not only contribute to people's donation behavior directly, but also can indirectly contribute to donation behavior by reinforcing perceived social responsibility and enhancing subjective social status. It can be seen that the mediating effect of language proficiency on donation behavior is through two mechanisms: altruistic mechanism is that language proficiency promotes donation behavior through perceived social responsibility; reciprocal mechanism is that language proficiency promotes donation behavior through subjective social status.

### 5.2. Theoretical implications

This study makes certain academic contributions and application value. From the standpoint of linguistics, this research offers a fresh analysis of the influence of Mandarin proficiency on an individual's donation behavior. It also investigates the mechanism and means by which Mandarin proficiency affects individual donation behavior using the mediation effect test. New theoretical foundations and concepts for the sociology of language are also provided by the literature that supplements behavior research.

Firstly, it broadens the newly created discipline of linguistic sociological research. This study adopts a novel strategy, starting from the viewpoint of a prosocial theory and extending the research on the consequences of language competence. Previous studies largely concentrated on the economic benefits and health effects of language on individuals (Dustmann and Fabbri, 2003; Tam and Page, 2016). The field and study subjects for the sociology of language research are expanded by accumulating the prosocial impact of language ability and concentrating on the influence of language ability on individual donation behavior at the level of the social importance of individual behavior.

Secondly, it broadens the study outlook on the variables that influence donating behavior. Past studies have produced a plethora of empirical studies to explain the influencing variables of individual donation behavior, including demographic traits, psychological qualities, and external environment, from the two views of individualism and structuralism. The ability endowment elements that influence individual donation behavior have, however, received little attention in the literature. Individual ability, such as linguistic skill, is a critical motivator for individuals to adopt long-term donation behavior.

Finally, this study examines the impact of language proficiency on donation behavior and its specific pathways. It deepens the research on the mechanism of individual donation behavior and enriches the theoretical research system of donation behavior. On the one hand, from the theoretical perspectives of altruism and reciprocity, this study has constructed the mixed mediating mechanism of perceived social responsibility and subjective class identification in language proficiency and donation behavior. On the other hand, Ability-motive Model provides a new perspective for the dynamic meaning construction of donation behavior, language ability promotes the explanatory path of donation behavior by strengthening the prosocial motives of perceived social responsibility and subjective class identity. The theoretical model of language proficiency-prosocial motives-donation behavior is constructed.

### 5.3. Practical implications

Practically speaking, the findings of this study not only offer a stronger foundation for promoting and popularizing Mandarin and for bolstering the development of national language skills, but also offer a new point of reference for interventions to raise the level of public donation behavior.

Firstly, Mandarin proficiency has a significant prosocial impact and both direct and indirect effects on an individual's donating behavior. It is clear that increasing national common language education, raising the quantity and quality of national common language popularization, fostering a positive linguistic climate, and boosting national language institutional capacity are all effective approaches to raising public donations. To achieve this, high-quality national common language enhancement should be applied at all levels and types of schools. At the same time, we should focus on the use of new media and integrated media, strengthen the impact of missionary education through technological empowerment, encourage the development of language and culture at various levels of society, and strengthen the national language. Increased cultural knowledge and proficiency in the mother tongue.

Secondly, it's essential to take into account the characteristics of donating behavior as well as the Mandarin proficiency of various groups. The impacts of Mandarin proficiency on willingness to donate also have different effects in different groups. This needs to optimize the Mandarin learning plans and pathways, boost the usefulness of the Mandarin effect, and maximize the prosocial effect of language ability.

Finally, in addition to the direct prosocial effect, the ability to speak Mandarin will also significantly strengthen people's sense of perceived social responsibility and improve their subjective social status, which is both crucial for maintaining national unity and realizing the peaceful and stable development of the nation and society. Moreover, the results of the control variables analysis have also provided a new perspective to prompt people to make donations. On the one hand, many factors such as demography, economy, and society have an effect on individuals' acts of charitable giving, which means that prompting citizens to donate is a systematic project, and the government needs to develop relevant policies to encourage people to do so.

### 5.4. Limitations

This study still has certain flaws because of the limits of secondary data indicators, even though the direct use of CGSS data can make the survey sample accurately reflect the entire population. To start with, language ability is a complicated idea. It also includes measuring its dimensionality and the language's substance. Language proficiency and donation behavior cannot be thoroughly multidimensionally analyzed in this study. Additionally, this study only has looked at the relationship between Mandarin competence and donation behavior; it did not look at dialect or foreign language proficiency, which are important markers of a person's level of ability in a particular language. Therefore, future research can provide a comparative analysis viewpoint to evaluate and examine the influence mechanism of various types and levels of language abilities on various types of donation behaviors from a more subdivided dimension.

Secondly, individuals' Mandarin proficiency and donation behavior may change as time goes by and technology develops, the CGSS2012 has measured these two variables. Although there is no denying that the findings have provided a guideline for us to think about the effect of linguistic ability on donation, it is necessary to be cautious when generalizing its conclusions since the data was collected long ago. To determine whether a linguistic ability's effect on contribution behavior is consistent over time, further study will need to update the survey data. In addition, future studies can also compare and contrast the variables between linguistic proficiency and donation behavior in other circumstances employing samples of individuals from other regions and countries.

Finally, the study only has explored the mediating effects of perceived social responsibility and subjective social status because it had a limited number of variables to work with, and the proportion of mediating effects was low. However, there may be multiple mediating variables between language ability and donation behavior. Research on the prosocial effect mechanism of language ability can be improved by incorporating more and more significant mediating and moderating variables in the future. This will further enhance the unique influence of language ability on donation behavior.

## Data availability statement

Publicly available datasets were analyzed in this study. The datasets for this study can be found in the Chinese General Social Survey (CGSS) (http://cgss.ruc.edu.cn/). Please see: http://cgss.ruc.edu.cn/ for more details.

## Author contributions

QW wrote the literature review and performed the statistical analysis. LY performed and organized the theoretical background and completed the manuscript. RZ developed research hypotheses and revised the overall manuscript. All authors contributed to the article and approved the submitted version.
